# Exploring Oxygen Therapy as a Supporting Treatment for Asthma: Current Insights and Perspectives

**DOI:** 10.3390/ijms27010024

**Published:** 2025-12-19

**Authors:** Michał Zwoliński, Adrian Hovagimyan, Jakub Ignatowicz, Marta Stelmasiak, Aneta Lewicka, Tomasz Szopiński, Łukasz Szymański, Justyna Bień-Kalinowska, Bałan J. Barbara, Sławomir Lewicki

**Affiliations:** 1 Institute of Outcomes Research, Maria Sklodowska-Curie Medical Academy in Warsaw, Pl. Żelaznej Bramy 10, 00-136 Warsaw, Poland; michal.zwolinski@uczelniamedyczna.com.pl (M.Z.); justyna.bien-kalinowska@uczelniamedyczna.com.pl (J.B.-K.); slawomir.lewicki@uczelniamedyczna.com.pl (S.L.); 2 University Clinical Hospital in Opole, al. Witosa 26, 45-401 Opole, Poland; adrianhovagimyan8@gmail.com; 3 Faculty of Medical Sciences and Health Sciences, Casimir Pulaski University of Radom, Chrobrego 27 St., 26-600 Radom, Poland; panjakubignatowicz@gmail.com; 4 Department of Dietetics, Institute of Human Nutrition Science, Warsaw University of Life Sciences, Nowoursynowska 159c St., 02-776 Warsaw, Poland; marta_stelmasiak@sggw.edu.pl; 5 Military Centre of Preventive Medicine Modlin, 05-100 Nowy Dwór Mazowiecki, Poland; anet.lewicka@gmail.com; 6 Institute of Clinical Sciences, Maria Sklodowska-Curie Medical Academy in Warsaw, Pl. Żelaznej Bramy 10, 00-136 Warsaw, Poland; tomasz@urologia.waw.pl; 7 Department of Molecular Biology, Institute of Genetics and Animal Biotechnology, Polish Academy of Sciences, 05-552 Magdalenka, Poland; l.szymanski@igbzpan.pl; 8 Department of Environmental Threat Prevention, Allergology and Immunology, Faculty of Health Sciences, Medical University of Warsaw, Pawińskiego 3c, 02-106 Warsaw, Poland

**Keywords:** asthma, oxygen therapy, hyperbaric oxygen therapy (HBOT), immune system, immunomodulation

## Abstract

Asthma is a chronic inflammatory disorder of the airways affecting over 260 million people worldwide and remains a major clinical and socioeconomic challenge. Despite substantial advances in pharmacological management (including inhaled corticosteroids, β_2_-agonists, leukotriene receptor antagonists, and biologic therapies), many patients continue to experience uncontrolled symptoms or corticosteroid resistance. This persistent unmet need has prompted interest in adjunctive and alternative treatment strategies. Oxygen therapy during asthma exacerbations and worsening pulmonary obstruction is a standard life-saving procedure. However, various forms of oxygen therapy are being considered for long-term treatment to reduce the number of exacerbations. Experimental and preliminary clinical data indicate that oxygen therapy may offer multiple benefits, including improved oxygenation, anti-inflammatory effects, reduced oxidative stress, modulation of nitric oxide signaling, enhanced immune responses, and promotion of angiogenesis. These mechanisms may collectively alleviate airway inflammation and improve pulmonary function. Nevertheless, clinical evidence on hyperbaric oxygen therapy (HBOT) in asthma remains limited. Existing small-scale studies suggest its safety but provide inconclusive results regarding its efficacy. Potential adverse effects such as barotrauma, oxygen toxicity, and transient anxiety necessitate careful patient selection and standardized treatment protocols. Further large-scale, randomized controlled trials are required to determine the therapeutic value of HBOT and to define its role as an adjunctive therapy in the comprehensive management of asthma.

## 1. Introduction

Asthma is a major global health concern that affects populations across all regions and socioeconomic levels.

**Figure 1:**
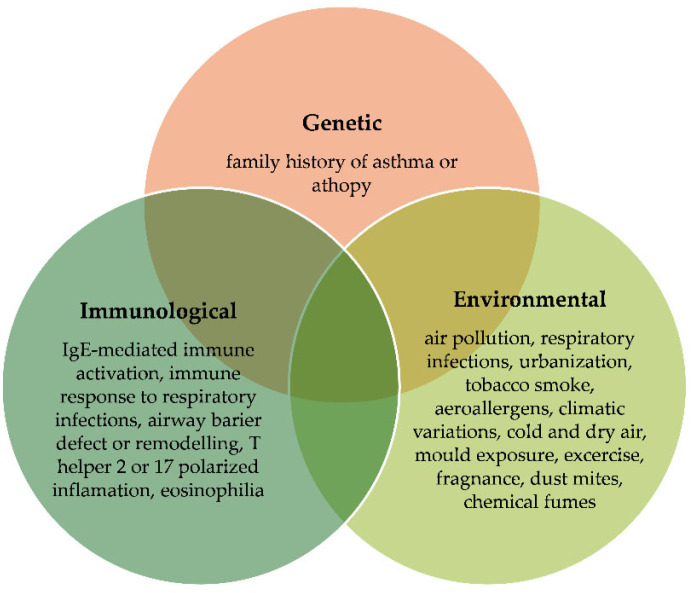
Asthma triggers as a combination of genetic, environmental and immunological factors.

According to 2021 data, approximately 260 million people worldwide are affected by the disease [1]. Asthma is a multifactorial disease influenced by genetic, environmental, and immunological factors and their complex interactions [2]. Its major clinical phenotypes include atopic/allergic, non-allergic, infection-related, and aspirin-exacerbated respiratory disease (AERD), and childhood pre-asthma syndrome [3]. Among these, atopic or allergic asthma is the most common [4], affecting up to 80% of allergic children and more than 50% of allergic adults [5]. Non-allergic asthma, which lacks clear sensitization to allergens, occurs in 10–33% of patients [6]. The most frequent asthma triggers are presented in Figure 1. Severe asthma affects less than 10% of asthma patients, but it generates enormous healthcare costs. It is relatively resistant to conventional treatment and is associated with poorer treatment outcomes, leading to many complications [7]. Therefore, new methods of treatment are constantly being sought that would enable the disease to be controlled. One of the possible emerging therapies is hyperbaric oxygen therapy (HBOT), which has an inflammation-modulating effect [8]. This article is a systematic review that aims to evaluate the effect of oxygen therapy in patients with asthma.

## 2. Types and Phenotypes in Asthma

Asthma is a chronic, heterogeneous disease of the airways characterized by persistent inflammation and variable, usually reversible, airflow limitation. The inflammatory process leads to bronchial hyper-responsiveness, mucus hypersecretion, and airway remodeling, resulting in recurrent symptoms such as wheezing, cough, shortness of breath, and chest tightness. These symptoms fluctuate in intensity and may resolve spontaneously or in response to pharmacological treatment.

The key pathophysiologic feature of asthma is expiratory airflow limitation, largely attributable to airway narrowing caused by inflammation-induced structural and functional changes in the bronchial wall. Inflammatory infiltration includes eosinophils, neutrophils, lymphocytes, dendritic cells (DCs), innate lymphoid cells (ILCs), and mast cells, which release cytokines and growth factors driving bronchial hyper-reactivity, mucus overproduction, and tissue remodeling [9]. According to current diagnostic recommendations, asthma diagnosis is based on a characteristic clinical history combined with objective evidence of variable expiratory obstruction, most often confirmed by spirometry demonstrating reversibility after bronchodilator administration.

Several clinically recognized asthma phenotypes can be distinguished according to triggering factors and underlying mechanisms (Table 1). These phenotypes differ in immunopathological background, symptom profiles, and therapeutic responsiveness, highlighting the heterogeneity of asthma and the importance of personalized management strategies.

Across these phenotypes, common triggers include aeroallergens, cold or dry air, physical exercise, mold exposure, tobacco smoke, respiratory infections, and strong chemical odors or airborne toxins. Environmental and occupational exposures remain central determinants of disease exacerbations and represent key targets for public health intervention.

## 3. Social Aspect of Asthma

The prevalence of asthma varies considerably among countries: in Australia, approximately 21% of the population are affected [11], whereas in Poland, around 13% of individuals have been diagnosed with asthma [12]. Among Polish patients, mild asthma accounts for 14.89% of cases, moderate for 49.36%, and severe for 35.74%, highlighting a substantial clinical and economic burden [13]. In low- and middle-income countries, asthma is also widespread; for instance, its self-reported prevalence among adults reaches 11.1% in Sri Lanka [14] and 8% in Iran [15]. In the pediatric population, asthma prevalence averages around 10%, with the highest rate reported in Australia (28%) [16].

During the late twentieth century, particularly between the 1970s and 1980s, asthma prevalence rose sharply. For example, in South Wales the rate among 12-year-old children doubled from 6% to 12% between 1973 and 1984 [17]. The global upward trend persisted into the 1990s but has since stabilized or modestly declined in several regions. The distribution of asthma by sex varies with age: in childhood, the disease is more common in boys (11.9%) than in girls (7.6%), whereas after puberty women are more frequently affected (9.6% vs. 6.3%) [18]. This reversal is attributed to hormonal influences on immune regulation, particularly the Th2-based effects of estrogen and progesterone.

Between 1980 and 1996, both the absolute number and the rate of asthma cases increased, accompanied by a rise in asthma-related mortality, which drew global attention to the disease [19]. Despite more recent improvements in prevention and management, asthma remains a leading cause of morbidity worldwide. The Global Burden of Disease Study (1990–2021) reported a 9.34% decline in total case numbers and a 40.01% reduction in the age-standardized prevalence index. Nevertheless, the Global Asthma Report (2025) estimates that one in ten children and one in fifteen adolescents currently live with asthma. High-income countries demonstrate high incidence but low mortality, reflecting efficient healthcare and early diagnosis, while low-income regions show lower incidence yet disproportionately higher mortality [1].

Asthma extends far beyond its medical implications, exerting a profound influence on patients’ overall quality of life, productivity, and psychosocial well-being. The disease affects not only physical health but also occupational functioning, emotional stability, and family dynamics. In addition to chronic symptoms and limitations in daily activities, individuals with asthma often experience reduced self-esteem and elevated psychological distress, contributing to a diminished overall quality of life [20]. From an occupational perspective, asthma is a well-established risk factor for nonattendance and decreased work productivity. Affected individuals are more likely to take recurrent sick leave, typically five or more within a single year, and demonstrate increased rates of presenteeism, defined as attending work while feeling unwell, which can result in reduced performance while at work [21].

Compared to healthy employees, asthma patients exhibit a higher probability of short-term medical leave lasting up to three consecutive days [22]. This reduced productivity translates into substantial economic losses. In the United States, annual per-patient healthcare expenditures across all age groups are estimated to be USD 700–2200, and the total annual national cost is approximately USD 21 billion [23]. Recent analyses indicate that workers with asthma incur significantly higher incremental medical expenses and experience more frequent occupational disability compared with their non-asthmatic counterparts [24].

The psychosocial burden of asthma is equally significant. Individuals with asthma exhibit increased rates of anxiety, embarrassment, and social withdrawal, which can impair adherence to treatment regimens and negatively affect disease control [25]. A 2021 meta-analysis demonstrated that individuals with asthma are at higher risk of experiencing anxiety compared to the general population [24]. Moreover, the impact of asthma extends beyond patients themselves, affecting their families as well. Parents of children with asthma often report diminished quality of life, increased stress levels, sleep disturbances, and a greater risk of depression, all of which may indirectly impair the child’s health and overall well-being [26].

## 4. Immune Mechanisms of Asthma and Their Modulation Using Oxygen Therapy

In recent years, asthma has been classified into two major endotypes: Type-2-high and Type-2-low asthma. Type-2-high asthma is characterized by elevated secretion of interleukins IL-4, IL-5, and IL-13, increased eosinophil counts, and higher total immunoglobulin E (IgE) levels. This endotype encompasses both allergic and eosinophilic asthma. In contrast, Type-2-low asthma is defined by the absence of Type-2 inflammatory biomarkers and eosinophilic elevation. It includes neutrophilic, paucigranulocytic (defined by the absence of sputum eosinophilia or neutrophilia), and obesity-related asthma phenotypes. Among these, the paucigranulocytic subtype is most commonly observed in individuals with well-controlled disease [2,27]. Importantly, both hypoxia and oxidative stress vary between phenotypes, and these factors are directly modulated by oxygen-based therapies [28].

The lung airway epithelium plays a key role in the initiation and orchestration of the immune response to various environmental factors. Inhaled agents such as allergens, pollutants, and respiratory viruses are sensed by epithelial cells through a broad spectrum of pattern recognition receptors (PRRs), including toll-like receptors (TLRs), nucleotide-binding oligomerization domain (NOD)-like receptors (NLRs), C-type lectin receptors (CLRs), retinoic acid-inducible gene I (RIG-I)-like receptors (RLRs), protease-activated receptors, and purinergic receptors [29]. Upon activation, airway epithelial cells release inflammatory cytokines, chemokines, and other mediators that alert and activate various immune cells. Among these epithelial-derived cytokines, often referred to as alarmins, are thymic stromal lymphopoietin (TSLP), interleukin-25 (IL-25), and interleukin-33 (IL-33). TSLP, IL-25, and IL-33 activate type 2 innate lymphoid cells (ILC2), which in turn produce Th2-associated cytokines such as IL-4, IL-5, and IL-13, thereby driving type 2 lung inflammation [30,31]. These alarmin-driven pathways increase oxygen consumption and contribute to localized hypoxia in inflamed airway tissue, which creates a biological rationale for therapies that raise tissue oxygen tension and modulate downstream inflammatory signaling [32].

TSLP is regarded as a key regulator of type 2 immune responses at the respiratory barrier. Beyond its role in activating Th2 cells and ILC2, it also acts on mast cells and enhances dendritic cell function by inducing the expression of costimulatory molecules [33]. Elevated TSLP expression has been detected in the airway epithelium and bronchoalveolar lavage (BAL) fluid of individuals with asthma, where it correlates with disease severity and progressive loss of lung function [34]. Hyperoxia has been shown to reduce several proinflammatory cytokines associated with epithelial activation, and this suggests that oxygen therapy might attenuate TSLP-dependent amplification of airway inflammation [28].

IL-33 plays a critical role in a range of inflammatory processes, including allergic asthma, through signaling via its receptor ST2. Binding of IL-33 to ST2 stimulates ILC2, mast cells, basophils, and Th2 cells, leading to the secretion of high levels of IL-5, IL-9, and IL-13 [35]. Also, IL-25, referred to as IL-17E, belongs to the IL-17 cytokine family. While other members of this family, such as IL-17A and IL-17F, are primarily associated with neutrophilic inflammation, proinflammatory cytokine induction, and type 1 immunity, IL-25 uniquely promotes type 2 immune responses, including eosinophilic inflammation and the excessive production of IL-4, IL-5, and IL-13 [36]. Moreover, IL-25 not only drives eosinophilic inflammation and airway hyper-responsiveness (AHR), but also contributes to structural airway remodeling, characterized by goblet cell hyperplasia, subepithelial collagen deposition, and angiogenesis [37]. Oxygen-based therapies which downregulate IL-1β, tumor necrosis factor α (TNF-α), and IL-6 may indirectly limit IL-33- and IL-25-mediated amplification of type 2 inflammation by reducing epithelial cell stress and cytokine release [28].

### 4.1. Mechanisms of Th2-High Asthma

Type 2 (T2) inflammation represents the predominant immunological pathway in asthma and is characterized by eosinophilic airway infiltration together with Th2-dependent overproduction of cytokines such as IL-4, IL-5, and IL-13. IL-4 and IL-13 activate B cells, leading to IgEproduction, which subsequently binds to the high-affinity IgE receptor (FcεRI) expressed on mast cells [37]. Mast cell-derived mediators, including leukotrienes (LTs), histamine, and interleukins, contribute to bronchoconstriction, airway inflammation, and structural remodeling across different asthma endotypes. Elevated IgE production has been correlated with increased levels of IL-5, IL-6, IL-17, and TNF-α [38]. Hyperbaric oxygen has been reported to decrease circulating IgE- and eosinophil-associated activity in allergic conditions, which suggests a potential modulatory effect on Th2-dominant pathways in asthma [39].

In addition, IL-4 induces mucus hypersecretion by activating the *MUC5AC* gene [40]. The principal sources of IL-5 are ILC2 and Th2 cells [41]. Patients with asthma display elevated IL-5 levels in both serum and bronchial biopsies [42]. IL-5 plays a central role in eosinophil biology, promoting their production, maturation, and recruitment to the lungs, while also stimulating mast cells to release histamine [43]. Reduction in IL-5-related eosinophil activation through oxygen-mediated decreases in proinflammatory cytokines has been observed in other allergic models and may translate to reduced airway eosinophilia during oxygen-based therapy [28].

Eosinophils themselves release a variety of mediators, including major basic protein (MBP), which enhances mast cell activation and further promotes histamine and leukotriene release. IL-13 contributes directly to airway pathophysiology by sensitizing airway smooth muscle to contractile stimuli, stimulating epithelial cells to secrete mucins, and inducing fibrotic remodeling processes [43,44]. By improving tissue oxygenation and lowering oxidative stress, HBOT may limit IL-13-mediated remodeling responses, which are enhanced under hypoxic conditions [45].

In addition to classical Th2-driven mechanisms, other T-cell subsets contribute to callergic airway disease, including IL-9-producing Th9 cells, as well as Th1 and Th17 cells. Th9 cells, through secretion of cytokines such as IL-9, IL-10, and IL-21, are implicated in amplifying Th2-associated lung inflammation, with IL-9 considered a particularly important mediator of asthma pathology [46]. IL-9 exerts pleiotropic effects on multiple immune cell types, including T cells, B cells, mast cells, and macrophages. It can promote Th2 inflammation by directly activating Th2 cells and enhancing mast cell recruitment and accumulation. Furthermore, IL-9 has been shown to activate Arg1+ interstitial macrophages, which secrete the chemokine C-C motif chemokine ligand 5 (CCL5). CCL5 subsequently recruits eosinophils, T cells, and monocytes into the lungs, thereby sustaining and propagating type 2 inflammation [46,47]. Oxygen therapy has been shown to enhance the production of IL-10, an anti-inflammatory cytokine which may counterbalance IL-9-driven inflammation and reduce downstream recruitment of inflammatory cells [48].

Conversely, regulatory T cells (Tregs) represent a specialized CD4+ T cell subset that plays a pivotal role in suppressing immune responses to maintain homeostasis and self-tolerance. Tregs may counteract asthma pathogenesis by inhibiting the activation and effector functions of a broad range of immune cells, including ILC2, mast cells, antigen-presenting cells, Th1/Th2/Th17 cells, eosinophils, neutrophils, and B cells [46]. Because HBOT increases IL-10 and reduces oxidative stress conditions known to support Treg function, oxygen-based therapies may indirectly enhance regulatory pathways in asthma [28].

### 4.2. Mechanisms of Th2-Low Asthma

Type-2-low asthma is characterized by aberrant immune responses mediated by Th17 and/or Th1 lymphocytes. CD4+ Th17 cells, a principal source of interleukin IL-17, play a central role in neutrophilic inflammation and airway remodeling and are implicated in corticosteroid resistance in asthma. Th1 cell activation drives the production of interferon (IFN)-γ and IL-2, while elevated levels of TNF-α, IL-6, IL-8, and IL-22 further promote neutrophilic inflammation [49,50,51,52]. HBOT has been shown to significantly reduce TNF-α and IL-6, which are core mediators of the Th17-dominated non-eosinophilic asthma phenotype, suggesting a mechanistic basis for its use in treating steroid-resistant disease [28].

IL-17 expression is significantly upregulated in lung tissue, BAL fluid, sputum, and peripheral blood of individuals with allergic asthma. IL-17 cytokines stimulate airway epithelial cells and fibroblasts to release neutrophil chemo-attractants, including CXCL1, CXCL5, and CXCL8, as well as granulocyte–macrophage colony-stimulating factor (GM-CSF), which collectively drive neutrophil recruitment into the airways. Moreover, IL-17A enhances airway smooth muscle contractility, migration, and proliferation, thereby contributing to AHR and airway remodeling—hallmark features of asthma [53]. Although asthma has traditionally been associated with Th2-driven eosinophilic inflammation mediated by eosinophils, mast cells, and Th2 lymphocytes, severe asthma is frequently accompanied by increased airway neutrophilia. Consequently, IL-17 signaling, which orchestrates neutrophil recruitment, represents a pivotal mechanism in asthma pathogenesis and a potential determinant of disease severity [54]. Oxygen therapy, through a reduction in oxidative stress, may interrupt IL-17-driven neutrophilic circuits, since reactive oxygen species (ROS) amplify IL-17 signaling and airway smooth muscle hyper-reactivity [45].

Persistent airway inflammation in asthma also promotes oxidative stress and dysregulated immune responses. Oxidative stress arises from excessive accumulation of reactive oxygen and nitrogen species (RONS) and/or impaired antioxidant defenses. In asthmatic airways, inflammation precedes oxidative injury, whereby RONS disrupt goblet cell integrity, leading to excessive mucus hypersecretion. These processes contribute to structural alterations in the bronchial wall (airway remodeling), which amplify inflammatory mediator release and exacerbate acute disease flares [55]. Importantly, RONS impair glucocorticoid receptor signaling, thereby inducing corticosteroid insensitivity and perpetuating proinflammatory signaling cascades in both immune and airway structural cells [56]. Both normobaric and hyperbaric oxygen have been shown to activate the Nrf2 antioxidant pathway and increase antioxidant enzymes, which may counteract oxidative-stress-driven steroid resistance in asthma [57].

Taken together, these mechanisms suggest that oxygen-based therapies may not influence all asthma endotypes equally. In T2-high eosinophilic asthma, where IgE-driven activation of mast cells and IL 4, IL 5, and IL 13 dominate, HBOT has been reported to reduce IgE levels and eosinophil activity in allergic conditions, which indicates that oxygenation may preferentially dampen type-2-cytokine-driven inflammation [39]. In contrast T2-low neutrophilic asthma is strongly associated with IL 17 TNF-α and IL 6 as well as high oxidative stress and corticosteroid resistance. These pathways are more directly modulated by oxygen therapy because HBOT and normobaric oxygen markedly reduce TNF-α, IL-1β and IL-6 and activate antioxidant Nrf2 signaling, which may be particularly beneficial in neutrophilic-steroid-resistant disease [58,59,60]. Therefore, oxygen therapy may have broader mechanistic relevance for T2-low asthma, yet it may still provide benefits in T2-high disease by reducing eosinophil-associated cytokine activity and improving tissue oxygenation.

In conclusion, the current evidence shows that HBOT can acutely reduce proinflammatory cytokines (e.g., IL-1β, TNF-α), and anti-inflammatory shifts are generally short-lived and mainly occur immediately after the first HBOT session or shortly after each session, not necessarily lasting between sessions [61]. There is no evidence that HBOT produces sustained baseline reductions in inflammatory markers or induction of long-term immunomodulation. Because asthma is a chronic inflammatory airway disease, such transient immunological effects are unlikely to result in meaningful or durable clinical improvements. The long-term relevance of these short-term cytokine changes remains uncertain and requires further investigation.

**Figure 2:**
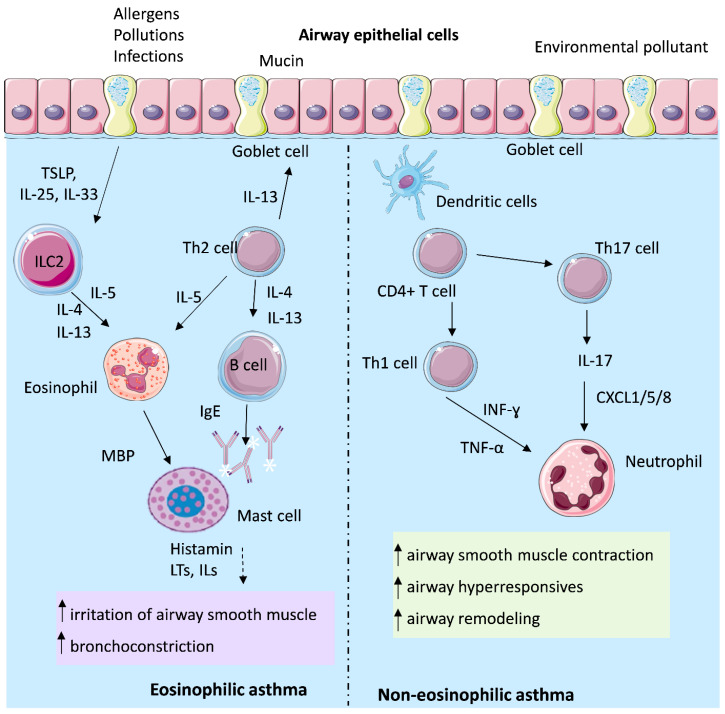
Pathogenesis of T2-high and T2-low asthma. “Type 2” or “T2-high asthma” is heavily driven by eosinophils, mast cells, and T-helper 2 (Th2) cells. These components release interleukin-4 (IL-4), interleukin-5 (IL-5), and interleukin-13 (IL-13), which activate B cells to produce IgE. The “T2-low asthma” immune response is associated with an inflammatory profile characterized by neutrophilic inflammation. This type of asthma is driven by T-helper 1 (Th1) and T-helper 17 (Th17) immune responses. ↑—increase; TSLP—thymic stromal lymphopoietin, LTs—leukotriens, ILs—interleukins, TNF-α—tumor necrosis factor α, IFN-γ—interferon gamma.

The pathogenesis of T2-high and T2-low asthma is illustrated in Figure 2.

## 5. Standard and Alternative Treatment of Asthma

**Figure 3:**
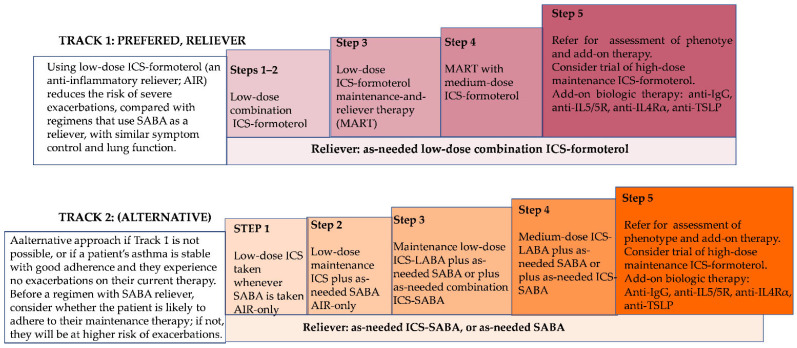
Two tracks of asthma treatment for adults and adolescents according to *Global Initiative for Asthma Guidelines 2024: An Update*.

The most effective therapeutic agents for asthma, and those recommended as first-line treatment for both adults and children with persistent asthma, are inhaled corticosteroids (ICSs). ICSs play a crucial role in controlling airway inflammation and preventing symptoms, while generally exhibiting fewer side effects compared to bronchodilators. A common approach to asthma management involves the use of β_2_-adrenergic receptor agonists, which exert both short- and long-term effects. In the short term, these agents alleviate acute asthma symptoms; however, their long-term use has been associated with an increased risk of hospitalizations and intubations [62]. According to the Global Initiative for Asthma (GINA 2024) [63], asthma management follows a stepwise approach that prioritizes ICSs in combination with bronchodilators rather than short-acting β_2_-agonists (SABA) alone. SABA monotherapy is no longer recommended, as it may increase the risk of severe exacerbations and even asthma-related mortality. Long-acting β_2_-agonists (LABAs) are typically prescribed in combination with ICSs for patients with moderate-to-severe asthma, as they help relax airway smooth muscles and improve airflow. Low and medium doses are sufficient in most patients, while high-dose ICSs are reserved for severe or uncontrolled disease. For patients who experience adverse effects from ICSs, treatment with antileukotrienes may be considered as an alternative therapeutic option [64]. Leukotriene receptor antagonists (LTRAs) such as montelukast are useful adjuncts in aspirin-exacerbated respiratory disease (AERD) and in patients with concomitant allergic rhinitis. Their efficacy is less pronounced than that of ICSs but they may improve adherence, particularly in children, due to their oral administration. In certain cases—particularly among monosensitized individuals—allergen immunotherapy (AIT) has shown potential efficacy and may be combined with other treatment modalities to enhance clinical outcomes. In cases of severe asthma, biologic therapies have become an important component of management [65]. Currently, several monoclonal-antibody-based treatments are available: omalizumab (anti-IgE); mepolizumab and reslizumab (anti-IL-5); benralizumab (anti-IL-5 receptor); dupilumab (anti-IL-4Rα); and tezepelumab (anti-TSLP) [66]. These biologics are particularly effective in patients with specific asthma phenotypes. The therapeutic effects of biologics vary depending on the agent used: mepolizumab and benralizumab have been shown to improve lung function (increase in forced expiratory volume in the first second (FEV_1_)); mepolizumab, benralizumab, and reslizumab reduce corticosteroid requirements; and mepolizumab and benralizumab enhance asthma control, as reflected by improved Asthma Control Test (ACT) scores [67,68]. Figure 3 includes a clear, step-by-step summary of the standard therapeutic protocols for asthma according to widely used international guidelines (e.g., GINA 2024). It includes treatment steps, pharmacological classes, and mechanisms of action [63]. The figure shows the main options for ongoing treatment for adults and adolescents as two treatment “tracks”. The key difference is the medication that is used for symptom relief. In Track 1 (preferred), the reliever is as-needed low-dose ICS–formoterol, and in Track 2, as-needed SABA or as-needed ICS-SABA. Track 1 is the preferred approach recommended for adults and adolescents, because using low-dose ICS–formoterol (an anti-inflammatory reliever [AIR]) reduces the risk of severe exacerbations, compared with regimens that use SABA as a reliever, with similar symptom control. The treatment regimen is simpler, with patients using a single medication as a reliever, and for maintenance treatment if prescribed, across treatment steps. With the AIR approach, when a patient at any treatment step has asthma symptoms, they use low-dose ICS–formoterol in a single inhaler for symptom relief. In Steps 1–2, this provides their anti-inflammatory therapy. In Steps 3–5, patients also take ICS–formoterol as their daily maintenance treatment; this is called “maintenance-and-reliever therapy” (MART). In Track 2, the reliever is as-needed SABA or as-needed ICS-SABA. This is an alternative approach if Track 1 is not possible, or if a patient’s asthma is stable with good adherence and they experience no exacerbations on their current therapy. In Step 1, the patient takes a SABA and a low-dose ICS together for symptom relief when symptoms occur. In Steps 2–5, SABA (alone) or combination ICS-SABA is used for symptom relief, and the patient takes maintenance ICS-containing medication regularly every day.

Despite advances in pharmacological therapy, a variety of alternative treatments for asthma are employed globally. While some alternative treatments for asthma have shown potential benefits, the overall evidence does not strongly support their effectiveness compared to traditional treatments. The literature describes methods such as using herbs and diet modifications to improve immunomodulation and reduce inflammation; performing physical and breathing exercises to improve lung function and quality of life; administration of oral bacterial lysates to improve immunity; use supplements of magnesium as an anti-inflammatory and bronchodilating agent; or usage of various off-label drugs, such as diuretics, anticoagulants, and macrolides. Additionally, the potential risks and side effects associated with alternative treatments highlight the need for further research to establish their safety and efficacy. Although these approaches have not been incorporated into official asthma management guidelines, numerous case reports describe clinical improvement following their use. The alternative treatment methods identified in this literature review are summarized in Table 2.

Although the modern treatment of asthma has evolved toward a personalized and phenotype-driven or disease-severity-based approach, there are still many patients who do not respond to the implemented treatment. In many cases the improvement after the prescribed treatment is not permanent. Therefore, there is a need to continuously develop new methods of treating asthma to induce sustained remission, minimize the number of exacerbations, decrease doses of used drugs to limit side effects, and improve the quality of patients’ lives.

## 6. Oxygenation in Asthma

One of the ways to improve the health condition of patients with asthma is the use of oxygen-based therapies. Oxygen administration in severe asthma exacerbations is a standard life-saving procedure to increase saturation. This paper is focused on complementary methods of asthma management that use oxygen. Oxygen therapy can be applied in various types of treatments, as it not only supports breathing but also has a broader physiological impact. Research suggests that oxygen therapy may significantly modulate the immune system’s response, which makes it increasingly explored as a supportive method in the treatment of allergic diseases [100]. This growing interest in oxygen therapy highlights its potential to complement traditional asthma management strategies and improve patient outcomes. It is interesting to note that there is a prevailing belief that oxygen therapy has no side effects and is recommended for almost all respiratory diseases. Notably, an increasing number of companies are offering HBOT to individuals with asthma, claiming that the therapy supports the healing process [101,102]. However, the available sources discussing the effects of HBOT lack references to original research studies. Therefore, we decided to conduct a systematic review to evaluate the impact of oxygen therapy in patients with asthma.

There are generally two clinical approaches to oxygen therapy: normobaric oxygen administration and HBOT. The beneficial effects of oxygen therapy in asthma are summarized in Table 3.

### 6.1. Normobaric Oxygen Therapy

Oxygen therapy increases the concentration of oxygen in inhaled air, enhancing its diffusion into the alveoli and thereby improving gas exchange within the lungs. This process helps to prevent hypoxemia, which is a common feature in patients with asthma, and supports the optimal function of pulmonary and systemic organs. Several forms of oxygen therapy are available. Acute oxygen therapy is administered using mechanical ventilatory support, whereas passive oxygen therapy is designed for patients who are able to breathe spontaneously. The primary objective of oxygen therapy is to deliver oxygen at higher concentrations through a face mask or nasal cannula. Oxygen can be supplied from cylinders containing compressed or liquid oxygen, or from medical devices that generate oxygen continuously, such as oxygen concentrators.

Conventional oxygen therapy remains the first-line intervention during acute asthma exacerbations. The optimal protocol for its use continues to be investigated, as oxygen administration can influence gas exchange and carbon dioxide retention. A randomized study demonstrated that the use of high-concentration oxygen therapy led to a clinically significant increase in transcutaneous carbon dioxide (PtCO_2_) levels in patients experiencing severe asthma exacerbations. As a result, a titrated oxygen regimen is recommended for the management of severe asthma, in which oxygen is administered only to patients with hypoxemia and at a concentration sufficient to correct oxygen deficiency without inducing hyperoxemia [105]. Recent studies have indicated that high-flow nasal oxygen therapy may be superior to conventional oxygen therapy in reducing respiratory distress during the early phase of treatment. In children with moderate-to-severe asthma exacerbations unresponsive to first-line therapy, high-flow nasal oxygen therapy significantly improved respiratory comfort and reduced distress within the first two hours of treatment [106]. Additional research has confirmed that nasal high-flow oxygen therapy decreases the severity of dyspnoea and lowers respiratory rate in hypoxemic patients with acute severe asthma [107]. Furthermore, the application of non-invasive positive pressure ventilation (NPPV) has been explored in the management of acute asthma attacks. In one study, patients were divided into high-pressure and low-pressure groups. Both treatment approaches resulted in significant improvements in forced expiratory volume in one second (FEV_1_), oxygen saturation, and respiratory rate, with greater benefits observed in the high-pressure group. These findings suggest that NPPV, even when delivered at lower pressures, may be an effective adjunctive therapy in the acute management of asthma exacerbations [108].

High-flow oxygen therapy (HFOT) is commonly utilized in the management of acute asthma exacerbations. Recent findings suggest that it may also have potential benefits when used over an extended period owing to its anti-inflammatory effects. By decreasing the concentration of inflammatory mediators that are typically elevated in individuals with asthma, HFOT may contribute to a reduction in chronic airway inflammation, a lower rate of exacerbations, and improvements in lung function. A meta-analysis conducted by Haoyue Deng and colleagues demonstrated that HFOT was associated with improved asthma control and patient comfort. The analysis revealed a reduction in respiratory rate, a decrease in the sensation of dyspnea, greater comfort during treatment, and a decline in the partial pressure of carbon dioxide (PaCO_2_), particularly in pediatric patients [109]. These outcomes indicate that HFOT may serve as a valuable adjunctive approach to standard pharmacological therapy in asthma management. The observed effects of normobaric oxygen therapy in asthma are summarized in Table 4.

### 6.2. Hyperbaric Oxygen Therapy in Asthma

HBOT is a clinical intervention that involves the administration of nearly pure oxygen, approaching 100% concentration, under elevated atmospheric pressures, typically between two and three atmospheres absolute (ATA) [32]. This approach substantially enhances oxygen tension within the bloodstream and various tissues, including the subcutaneous layer, thereby improving tissue oxygenation [59]. Over recent years, HBOT has gained recognition as a therapeutic modality for numerous medical conditions. An expanding body of evidence, comprising not only case studies but also well-designed randomized, double-blind clinical trials, supports its beneficial physiological effects [110,111]. The therapy has been implemented in the management of diverse pathologies, including chronic and acute wound healing, thermal and radiation-induced burns, carbon monoxide intoxication, gas embolism, severe anemia, clostridial myonecrosis (gas gangrene), crush injuries, compartment syndrome, acute traumatic ischemia, decompression illness, arterial insufficiencies, intracranial abscesses, necrotizing soft tissue infections, osteomyelitis, and idiopathic sensorineural hearing loss [112,113,114,115].

The precise mechanisms by which HBOT modulates immune function remain inadequately elucidated. Empirical evidence suggests that HBOT exerts significant anti-inflammatory effects and facilitates tissue repair. Several investigations have documented reductions in C-reactive protein (CRP) and proinflammatory mediators, including IFN-γ, TNF-α, and interleukins IL-1, IL-1β, and IL-6, and enhancements in anti-inflammatory cytokine IL-10 production [48,116]. Due to its anti-inflammatory character, Jermakow et al. (2025) suggest that HBOT may serve as an effective adjunctive treatment for patients with cytokine storms [117]. Considering these immunomodulatory and regenerative properties, hyperbaric oxygen therapy represents a promising adjunctive approach for the management of allergic and inflammatory disorders.

As a result of HBOT, ROS production increases due to hyperoxia, but the increased concentration persists only briefly after treatment. Similarly, the concentration changes with successive cycles of therapy; at the beginning, a significant increase in ROS concentration in the blood is usually observed, but as the therapy progresses, this concentration gradually decreases [118]. Hyperoxia and the presence of ROS result in increased production of heat shock protein 1 (HO-1), glutathione S-transferase, and quinone oxireductase 1, protecting the cell from the harmful effects of free radicals [45].

A recent prospective study, primarily conducted in healthy adults (n = 88; mean age ~60 years), demonstrated that HBOT was well tolerated, with no patients reporting typical pulmonary oxygen toxicity symptoms such as cough, dyspnea, chest discomfort, or irritation. A small but statistically significant improvement in pulmonary parameters was observed, including FVC (+0.1 ± 0.38 L) and PEF (+0.5 ± 1.4 L/min). The therapy consisted of 60 HBOT sessions (90 min, 100% oxygen, 2 ATA, with 5 min air breaks every 20 min, 5 days per week) [119]. The effect of HBOT on asthma is poorly understood. Preliminary research on this topic was performed in 1987. The subjects were divided into three groups—healthy, with various diseases, and with allergy. They were treated with HBOT, with positive results, but these were small and lacked modern diagnostic criteria. This first published study, although it represents a basis for studying the effectiveness of HBOT in the treatment of asthma, has some methodological limitations (non-randomized designs, outdated endpoints, absence of spirometry-based phenotyping) [8]. Since that study was conducted, the idea of using HBOT in the treatment of asthma is still being researched [120]. The most commonly indicated benefits are related to better oxygenation of lungs and tissues, often deprived of oxygen in asthma, because chronic airway inflammation and bronchoconstriction limit oxygen exchange. A decrease in inflammatory mediators, which are usually increased in patients with asthma, serves to reduce chronic inflammation, decrease the number of exacerbations, and improve lung function.

**Figure 4:**
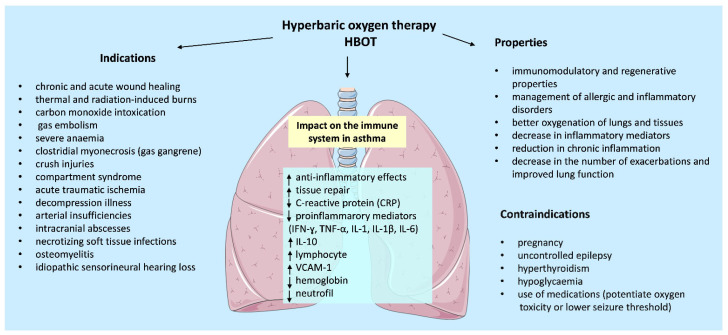
The beneficial impacts of normobaric and hyperbaric oxygen therapy (HBOT) in asthma. Summary of the biological effects of HBOT and its clinical applications. ↑—increase, ↓—decrease.

The described effects of various methods of oxygen therapy and high-pressure oxygenation in asthma are presented in Table 4 and Figure 4. The table summarizes various reports on various methods of oxygen therapy for asthma; however, when analyzing these data, one should remember the difference between therapy for respiratory failure in asthma exacerbations and oxygen therapy supporting conventional pharmacotherapy in the period between exacerbations—with the latter having a disease-modifying effect.

**Table 4:** Up-to-date summary of the effects of oxygen therapy in humans. We searched three main databases, PubMed, Google Scholar, and SCOPUS, for articles, with the following queries: oxygen therapy and asthma; HBOT and asthma; hyperbaric therapy and asthma. We present here the results from only original revised articles.

Normobaric Oxygen Therapy			
Group of Patients	Treatment	Significant Observations	References
106 patients with severe exacerbations of asthma	8 L/min via medium-concentration mask or titrated oxygen (to achieve oxygen saturations between 93% and 95%) for 60 min.	High-concentration oxygen therapy causes a clinically significant increase in Ptco2 in patients presenting with severe exacerbations of asthma	[105]
96 pediatric patients with acute exacerbations of asthma, 49 in the HCOT group and 47 in the TOT group	Prospective, randomized clinical trial comparing high-concentration (HCOT) to titrated oxygen therapy (TOT)	HCOT in pediatric asthma exacerbation leads to significantly higher carbon dioxide levels, which increases asthma scores	[121]
40 adult patients with moderate-to-severe asthma exacerbation, 20 in the HFNC group and 20 in the COT group	A randomized, double-blind pilot study comparing high-flow nasal cannula (HFNC, high-flow oxygen with a flow rate of 15–35 L/min (37 °C)) and conventional oxygen therapy (COT, flow rate of 2–5 L/min) in patients with asthma exacerbation	HFNC appears to be more effective than COT in reducing the dyspnea score within the first 2 h of treatment. In the HFNC-treated patients, FVC was improved from 2 to 24 h of treatment (25.7 ± 26.6%), while in the COT-treated patients, FVC increased significantly (9 ± 7%, *p* = 0.024, CI: 95%)	[122]
62 children (1–14 years) with moderate-to-severe asthma exacerbations to receive either high-flow nasal cannula or standard oxygen therapy	The initial flow rate depends on patient weight and clinical status. Depending on the degree of respiratory distress, PS, SpO2, and RR, clinicians are allowed to increase the flow rate if necessary up to the maximum that the patient can tolerate, without exceeding a flow of 2 L/kg/min for the first 10 ± 0.5 L/kg/min per kg above 10 kg	High-flow nasal cannula appears to be superior to conventional oxygen therapy for reducing respiratory distress within the first 2 h of treatment in children with moderate-to-severe asthma exacerbation	[106]
37 patients aged ≥ 18 years with acute severe asthma	Conventional oxygen therapy or nasal high flow for 120 min, adjusted from 30 to 60 L/min according to the participant’s level of comfort	Nasal high flow reduced the severity of dyspnea and respiratory rate in hypoxemic patients with acute severe asthma	[107]
44 patients with acute asthma of mild-to-moderate severity	Non-invasive positive pressure ventilation (NPPV) from 4 cm to 8 cm with H_2_O for 60 min	Without bronchodilators, the initial treatment with NPPV may improve pulmonary function and physical status with sustained efficacy, making it an additional therapeutic option for acute asthma in emergency or outpatient departments	[108]
Meta-analyses: 85 patients treated with high-flow oxygen therapy, and 90 patients with conventional oxygen therapy	High-flow oxygen therapy vs. conventional oxygen therapy	High-flow oxygen therapy decreased the dyspnoea score compared to conventional oxygen therapy	[109]
**Clinical studies of HBOT in asthma**			
11 men and 5 women	1.8 to 2.5 ATA O2 within a 60–90 min period. The number of exposures ranged from 1–4 for Group II to 10–15 for Group I and Group III	Generally, in allergic asthma, a positive therapeutic effect of hyperbaric oxygenation on was found, with no adverse effects	[8]
7	Over 20 sessions, HBOT was performed using 100% oxygen at a pressure of 2.0–2.4 atmospheres absolute (203–243 kPa) for 90 min, five times per week	Therapy was safe but there was no significant change in FEV1%, FVC%, or FEF25–75%	[123]
**Preclinical study HBOT in asthma**			
Mice (strain C57BL/6) were sensitized with ovalbumin (OVA) via intraperitoneal injection (day 1), then challenged with OVA inhalations on days 14–17 to induce allergic airway inflammation or asthma-like condition	HBOT was applied with 100% O_2_ at either 2 ATA or 3 ATA, for 60–90 min per session, over 4–5 days, either after sensitization or during inhalation challenges	HBOT at 3 ATA significantly reduced eosinophil infiltration in BALF and lowered BALF protein concentration and LDH activity (i.e., reduced lung tissue injury) compared to untreated OVA-challenged controls. 3 ATA HBOT significantly decreased serum total IgE levels, which may reflect modulation of systemic allergic response	[124]

### 6.3. Limitations of Using HBOT in Asthma

Although HBOT demonstrates potential therapeutic benefits, and is considered a safe and well-tolerated adjunctive treatment, certain findings raise important considerations regarding its safety in the context of asthma. The most frequently observed complications arise from barotrauma to air-containing cavities, such as the middle ear and paranasal sinuses, resulting from pressure differentials during compression and decompression [125]. These events are typically mild and reversible but can cause discomfort, tympanic membrane injury, or sinus pain if equalization techniques are inadequate. Claustrophobia and transient anxiety are also occasionally reported among patients undergoing chamber sessions.

At the immunological level, HBOT may have complex effects. Vascular cell adhesion protein 1 (VCAM-1), a glycoprotein whose expression is induced by proinflammatory cytokines and ROS, plays a critical role in transendothelial migration of leukocytes in asthma. IL-4 induces VCAM-1-mediated migration of eosinophils to airway tissues, contributing to airway inflammation and remodeling [126,127]. In a mouse model, Lee and colleagues [128] demonstrated that blocking VCAM-1 reduced recruitment of macrophages, neutrophils, and eosinophils to the lungs and ameliorated airway remodeling. Interestingly, HBOT may increase VCAM-1 and lymphocyte levels, accompanied by reductions in hemoglobin and neutrophil counts [129]. These findings suggest that the immunological consequences of HBOT in airway diseases remain incompletely understood and warrant further investigation, particularly in the context of asthma.

Ocular complications are another well-documented consequence of prolonged HBOT exposure. Repeated treatments may induce hyperbaric myopia, a transient refractive change caused by lens oxygenation and structural alteration, which typically resolves after discontinuation of therapy. In rare instances, extended or high-frequency exposure has been associated with cataract formation, keratoconus progression, or retinal vascular changes [130].

Patients with pre-existing pulmonary conditions such as chronic obstructive pulmonary disease (COPD), bronchial asthma, or upper respiratory tract infections warrant special consideration before initiating HBOT. Rapid pressure changes can exacerbate air trapping or induce bronchospasm, thereby increasing the risk of pulmonary barotrauma or pneumothorax. The Japanese Society for Hyperbaric and Undersea Medicine identifies active bronchial asthma as a temporary contraindication, recommending that treatment be deferred until respiratory symptoms are stabilized [131].

Certain physiological and medical conditions also represent relative or absolute contraindications to HBOT. These include pregnancy, uncontrolled epilepsy, hyperthyroidism, hypoglycemia, and the use of medications that potentiate oxygen toxicity or lower seizure threshold. In pregnant patients, case reports emphasize the need for cautious risk–benefit assessment due to potential fetal oxygen exposure, although HBOT has been applied successfully in select emergencies such as carbon monoxide poisoning [132]. According to clinical safety guidelines, all candidates for HBOT should undergo comprehensive screening for comorbidities and medication interactions before treatment initiation [133].

## 7. Conclusions

The literature suggests that oxygen therapy may exert beneficial effects through several mechanisms potentially relevant to asthma management, including improved oxygenation, anti-inflammatory action, reduced oxidative stress, enhancement of immune responses, promotion of angiogenesis, and modulation of nitric oxide pathways. Collectively, these mechanisms may contribute to symptom relief and improved pulmonary function in patients with asthma.

It is often hypothesized that HBOT may improve the clinical course and prognosis of asthma, and treatment protocols targeting this population are increasingly promoted in some clinical settings. However, current evidence supporting the efficacy of HBOT in asthma remains limited. Only a few small studies have reported potential benefits, and no randomized controlled trials have yet been conducted to confirm its effectiveness or safety. Consequently, the application of HBOT in asthma should be considered experimental and undertaken with caution. There is a clear need for well-designed, large-scale clinical trials to evaluate the therapeutic potential of HBOT in this indication. Future asthma management algorithms incorporating HBOT as an adjunctive intervention should be developed only on the basis of robust, evidence-based data derived from such studies.

## Figures and Tables

**Table 1:** Clinically recognized phenotypes of asthma and their main characteristics [10].

Phenotype	Primary Trigger or Mechanism	Key Clinical Features	Patient Characteristics
Allergic asthma	Exposure to environmental allergens. IgE-mediated immune activation	Common in childhood. Associated with other atopic diseases. Good response to corticosteroids and anti-IgE therapy	Typically younger patients; personal or family history of atopy; elevated IgE levels; positive allergen test
Cough-variant asthma (Corrao asthma)	Airway hyper-responsiveness without classic wheeze	Chronic cough as the sole or predominant symptom. May precede typical asthma	Often adults or children with persistent dry cough; normal lung auscultation; may show airway hyper-reactivity on testing
Exercise-induced asthma	Physical exertion leading to airway cooling and dehydration	Bronchoconstriction during or after exercise. Reversible with bronchodilators	Frequently affects adolescents and young adults engaged in sports; symptoms triggered specifically by exertion
Occupational asthma	Occupational asthma Inhalation of workplace sensitizers (e.g., isocyanates, latex, flour dust)	Symptoms improve outside the work environment. May require allergen avoidance or workplace modification	Adult workers exposed to specific occupational agents; symptom pattern linked to work shifts
Asthma–COPD overlap syndrome (ACOS)	Combination of asthmatic and chronic obstructive mechanisms	Persistent airflow limitation. Older age of onset. Reduced corticosteroid responsiveness	Typically older patients with a history of smoking or biomass exposure; mixed asthma-COPD features; frequent exacerbations

**Table 2:** Summary of alternative asthma treatment.

Alternative Asthma Treatment	Mechanism of Action	References
Magnesium (magnesium sulfate (MgSO_4_))	Anti-inflammatory and bronchodilating agent	[69]
Furosemide	Inhaled furosemide attenuates bronchoconstriction and asthma attacks	[70]
Heparins	Inhaled heparin reduces inflammation, thrombogenesis, atherogenesis, and cell proliferation in airways and reduces eosinophilic and lymphocytic counts in bronchoalveolar lavage (BAL) samples	[71]
Macrolide (azithromycin)	Reduces exacerbations and improves quality of life, and induces remission in both eosinophilic and non-eosinophilic asthma	[72]
Nitric oxide donors	Relax the muscles in the airways during an asthma crisis	[73]
Antioxidative drugs (N-acetylcysteine, Nrf2)	Improve small-airway function, decrease exacerbation frequency, and play a protective role against reactive oxygen species (ROS)	[74,75,76,77]
Antioxidant vitamins (vit. A, vit. E, vit. C), plant-based antioxidants	Block proinflammatory pathways and protect against oxidative damage	[78,79,80]
Vitamin D	Has an immunomodulatory effect and reduces risk of asthma exacerbation	[81,82]
Herbal therapy	Immunomodulatory, anti-inflammatory and bronchodilatory effects	[83,84]
	*Nigella sativa* (black cumin): Improves asthma control and pulmonary function, and reduces eosinophils	[85]
	*Crocus sativus* L. (saffron): Improves asthma symptoms, pulmonary function, and immunological parameters	[86]
	*Pinus maritima* (maritime pine): Improves asthma symptoms, and reduces levels of leukotrienes C4, D4, and E4	[87]
	*Curcuma longa* (curcumin): Induced asthma control, and decreases frequency of symptoms and nighttime awakenings	[88]
	*Echinacea*: Performs immunomodulatory activity and blocks ferroptosis	[89,90]
	*Fritillaria cirrhosa*: Inhibits M2 macrophage polarization, and exerts anti-asthmatic effects in murine models by reducing eosinophil numbers and suppressing Th2 cytokines (IL-4, IL-5, IL-13), IgE, and histamine production	[91]
	*Anemarrhena asphodeloides*: Regulates the arachidonic acid pathway and regulates mast-cell-mediated reactions	[92]
Dietary modifications	Incorporating antioxidant-rich foods, reducing saturated fat intake, emphasizing the consumption of plant-based foods, and maintaining a healthy weight reduce systemic inflammation and oxidation, and improve microbial composition	[93,94]
Acupuncture and massage	Modulate the immune system, reduce airway inflammation, induce bronchodilation, and regulate neurotransmitters involved in bronchial smooth muscle contraction; acupoint massage combined with ear-point-pressing beans has a good effect on the treatment of asthma remission and can effectively improve quality of life	[95,96]
Yoga, breathing techniques	Breathing techniques may improve breathlessness	[97]
Exercises, physical activity, pulmonary rehabilitation	Improve cardiorespiratory fitness and muscle strength, increasing forced vital capacity % (FVC%) pred and FEF25–75%	[98]
Oral bacterial lysate (OM-85)	Reduces the number of acute respiratory tract infections, prevents allergic inflammation by enhancing Treg cell proliferation and acetate production, and alleviates the course and length of exacerbations	[99]

**Table 3:** The possible positive effects of oxygenation in asthma.

Mechanism	Description	Relevance to Possible Asthma Management	References
Increased Oxygenation	Higher oxygen levels in blood and tissues	Alleviates hypoxia in inflamed airways	[32]
Anti-inflammatory Effects	Reduction in proinflammatory cytokines (e.g., TNF-α, IL-1β)	Reduces chronic airway inflammation	[28]
Anti-allergy Effects	HBOT decreases serum IgE levels and reduces eosinophil infiltration	Potentially reduces allergic inflammation	[39]
Reduction in Oxidative Stress	Modulation of ROS and RNS (reactive nitrogen species) production	Mitigates oxidative stress and airway hyper-reactivity	[45]
Enhanced Immune Response	Increased bactericidal activity of immune cells	Manages infections that exacerbate asthma	[103]
Promotion of Angiogenesis	VEGF (vascular endothelial growth factor) proliferation and enhanced fibroblast activity	Aids in tissue repair and reduces airway remodeling	[28]
Modulation of Nitric Oxide	Influence on nitric oxide pathways	Reduces airway constriction and improves airflow	[104]

## Data Availability

No new data were created or analyzed in this study. Data sharing is not applicable to this article.

## Abbreviations

The following abbreviations are used in this manuscript:

HBOT	hyperbaric oxygen therapy
AERD	aspirin-exacerbated respiratory disease
RSV	respiratory syncytial virus
DCs	dendritic cells
ILCs	innate lymphoid cells
NSAIDs	non-steroidal anti-inflammatory drugs
COPD	chronic obstructive pulmonary disease
IL-	interleukin
TNF-α	tumor necrosis factor alpha
PRRs	pattern recognition receptors
TLRs	toll-like receptors
NOD	nucleotide-binding oligomerization domain
RLRs	retinoic acid-inducible gene I like receptors
TSLP	thymic stromal lymphopoietin
BAL	bronchoalveolar lavage
ST2	receptor for the cytokine IL-33
AHR	hyper-responsiveness
IgE	immunoglobulin E
FcεRI	IgE receptor
LTs	leukotrienes
MBP	major basic protein
CCL5	C-C motif chemokine ligand 5
Arg1	arginase-1 enzyme
Tregs	regulatory T cells
CXCL1-, 5- 8-	C-X-C motif chemokine 5
GM-CSF	granulocyte–macrophage colony-stimulating factor
RONS	reactive oxygen and nitrogen species
SABAs	short-acting β_2_-agonists
LABAs	long-acting β_2_-agonists
ICSs	inhaled corticosteroids
LTRAs	leukotriene receptor antagonists
AIT	allergen immunotherapy
FEV	forced expiratory volume
ACT	asthma control test
ATA	atmospheres absolute
CRP	C-reactive protein
NPPV	non-invasive positive pressure ventilation
VCAM-1	vascular cell adhesion molecule-1
